# Supplementary material for: Quantifying compartment‐associated variations of protein abundance in proteomics data
Source: Mol Syst Biol. 2018 Jul 2;14(7):e8131. doi: 10.15252/msb.20178131 (PMC6056770; doi:10.15252/msb.20178131)
Supplement: Supplementary file 1 — Expanded View Figures PDF [file MSB-14-e8131-s001.pdf]

Expanded View Figures

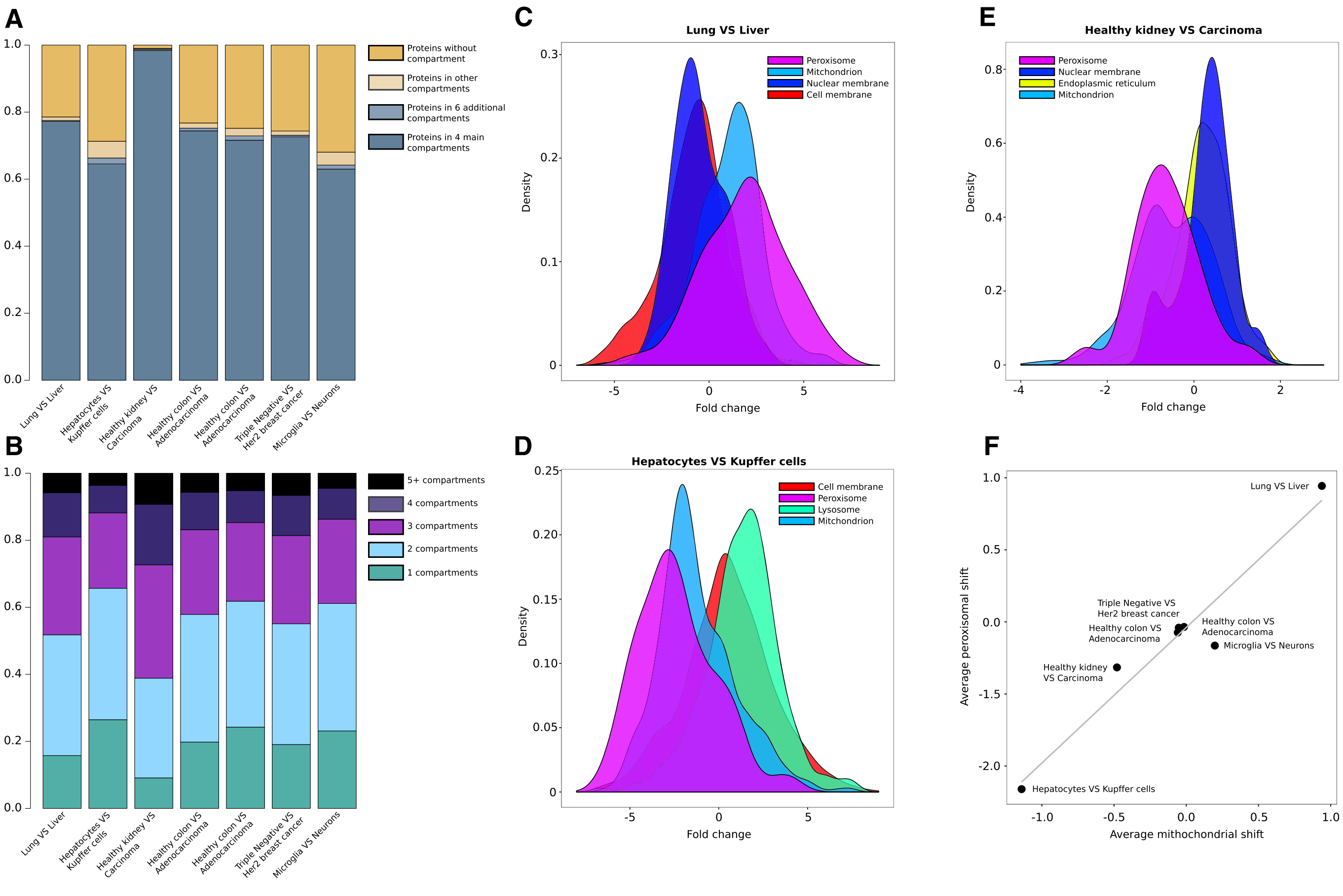

Figure EV1. Protein annotation and compartment fold change distribution for different proteomics datasets.

A Protein annotation in Gene Ontology (cellular component terms) for the seven datasets analyzed. Percentage of protein with (i) no GO annotation, (ii) any GO cellular component annotation, (iii) annotation to ten major cellular compartments (nucleus, cytoplasm, mitochondrion, extracellular space, endoplasmic reticulum, Golgi apparatus, cell membrane, nuclear membrane, lysosome, and peroxisome), and (iv) annotation to four major cellular compartments (nucleus, cytoplasm, mitochondrion, and extracellular space) are reported for each dataset.

B Percentage of proteins annotated to one, two, three, four, five, or more compartments in each dataset, in order (Geiger *et al*, 2012; Wiśniewski *et al*, 2012; Azimifar *et al*, 2014; Guo *et al*, 2015; Sharma *et al*, 2015; Wisńiewski *et al*, 2015; Tyanova *et al*, 2016).

C–E Density plot for the top four significant compartments (Mann–Whitney test $P < 0.05$) depicting the average fold change distributions for (C) lung vs. liver cells (Geiger *et al*, 2013), (D) hepatocytes vs. Kupffer cells (Azimifar *et al*, 2014), and (E) healthy kidney vs. renal carcinoma cells (Guo *et al*, 2015).

F Scatter plot of average abundance shift of mitochondrial proteins (*x*-axis) and peroxisomal proteins (*y*-axis) in seven different datasets (Geiger *et al*, 2012; Wiśniewski *et al*, 2012; Azimifar *et al*, 2014; Guo *et al*, 2015; Sharma *et al*, 2015; Wisńiewski *et al*, 2015; Tyanova *et al*, 2016); a gray line represents the line fitted using the resulting points (Pearson's $R = 0.97$, $P = 3.5 \times 10^{-4}$). Proteins shared between the two compartments (annotated as both mitochondrial and peroxisomal) were excluded for this analysis.

Data information: Related to Fig 1 and Dataset EV4.

 © 2018 The Authors

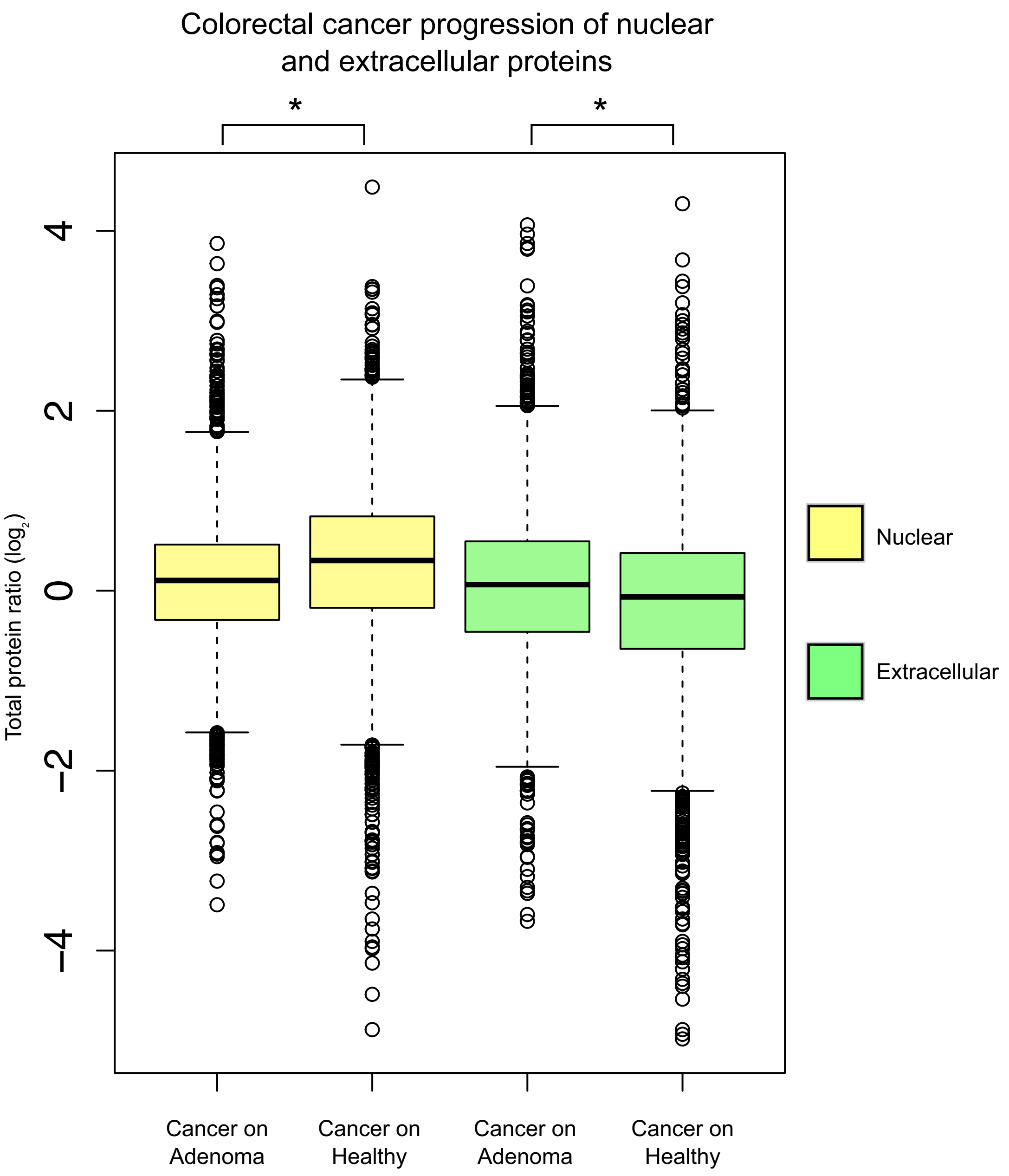

Figure EV2. Collective shifts of abundance for nuclear and extracellular proteins in colorectal cancer.

Cellular compartment shifts during colorectal cancer progression: cancer/healthy protein ratios are compared to cancer/adenoma protein ratios for 3,231 and 3,206 nuclear proteins, respectively (Mann–Whitney test *$P < 0.01$), and for 2,172 and 2,155 extracellular proteins, respectively (Mann–Whitney test *$P < 0.01$; Wiśniewski *et al*, 2015). The average ratio of the protein abundances between two conditions is represented by boxplots; significant comparisons are marked with stars. Related to Fig 1. Boxplots: the horizontal line represents the median of the distribution, the upper and lower limit of the box indicate the first and third quartile, respectively, and whiskers extend 1.5 times the interquartile range from the limits of the box. Values outside this range are indicated as outliers points.

© 2018 The Authors

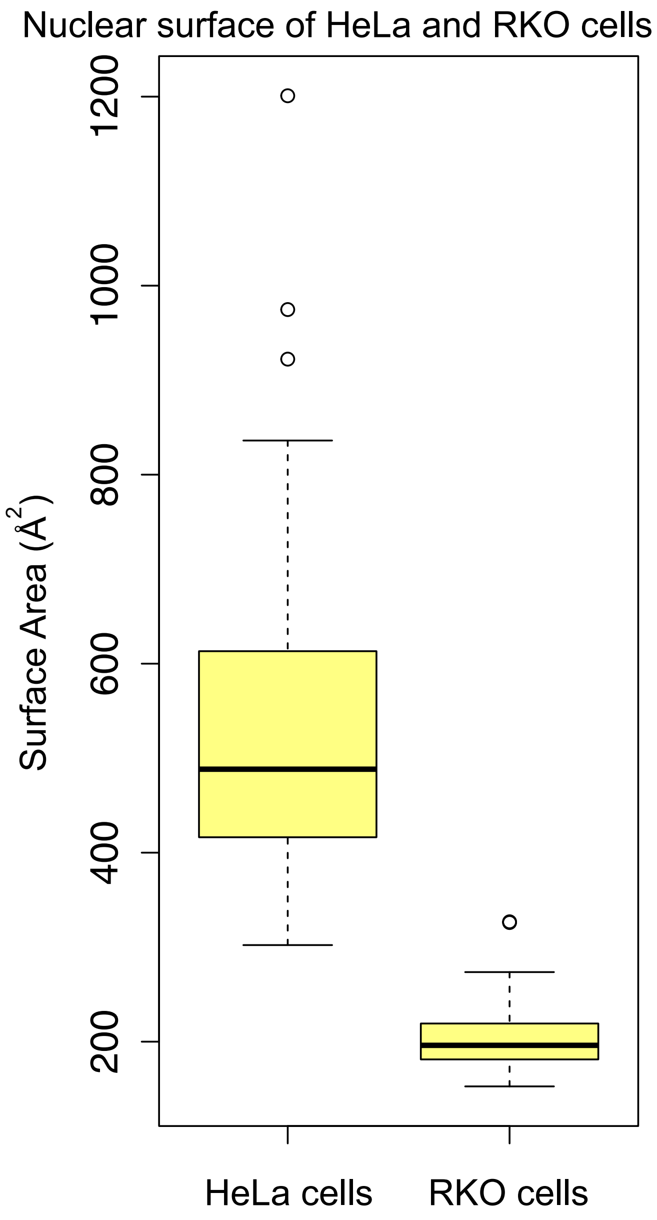

Figure EV3. Comparison of nuclear surface area of HeLa and RKO cells.

Distributions, represented as boxplots, of nuclear surface area ($Å^2$) of 44 HeLa and 41 RKO cells (Ori *et al*, 2013). HeLa nuclei are significantly larger than RKO nuclei (*t*-test $P = 2.5 \times 10^{-15}$). Nuclear surface area was estimated from the radius of isolated nuclei measured in phase contrast images, assuming a spherical shape. Related to Fig 2D and Dataset EV5. Boxplots: the horizontal line represents the median of the distribution, the upper and lower limit of the box indicate the first and third quartile, respectively, and whiskers extend 1.5 times the interquartile range from the limits of the box. Values outside this range are indicated as outliers points.

 © 2018 The Authors

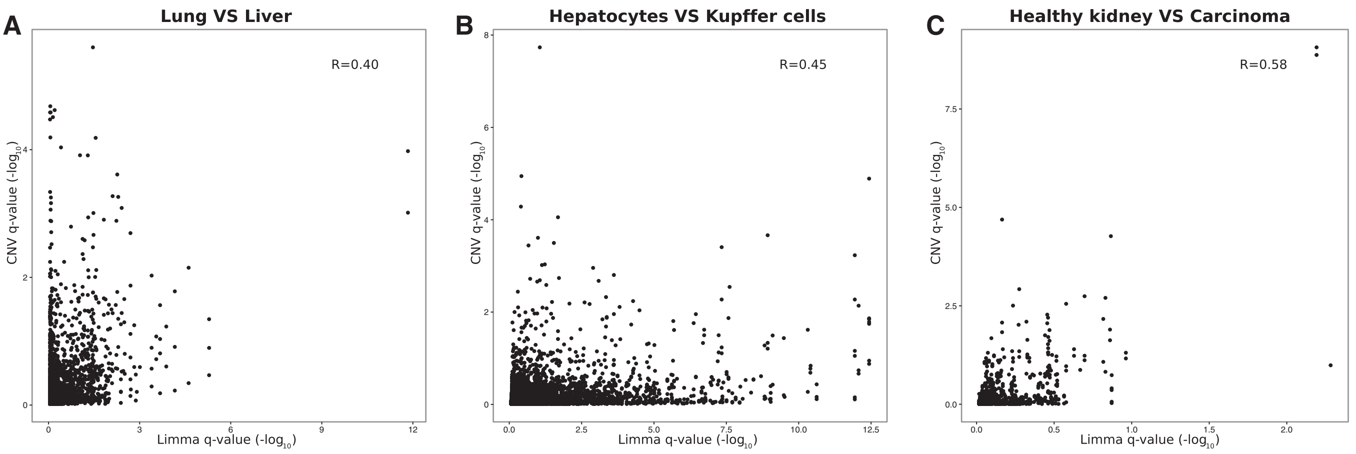

Figure EV4. Comparison of statistics obtained by standard differential expression (limma) and CNV approach applied to the same dataset.

A–C Scatter plots of *q*-values ($-\log_{10}$-transformed) associated to limma protein fold changes (*x*-axis) and CNV values (*y*-axis) for (A) lung vs. liver cells (Geiger *et al*, 2013), (B) hepatocytes vs. Kupffer cells (Azimifar *et al*, 2014), and (C) healthy kidney vs. renal carcinoma cells (Guo *et al*, 2015). Pearson's correlations are reported inside the plots. Related to Fig 2F and H and Datasets EV1–EV3.

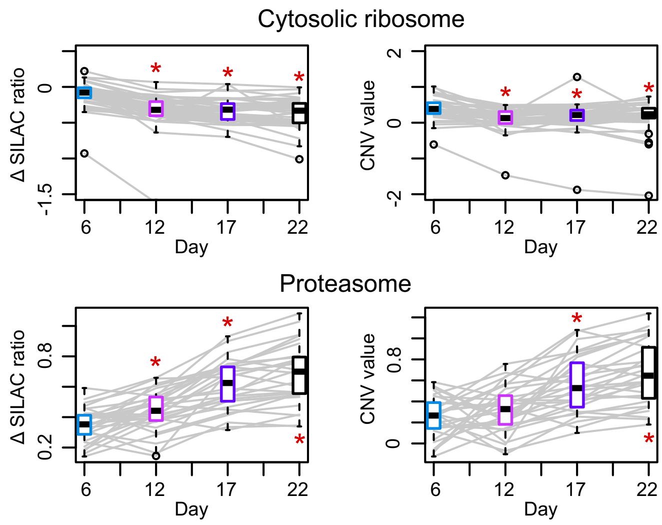

Figure EV5. Complex subunit variation during *Caenorhabditis elegans* aging.

Abundance variation of complex members of the cytosolic ribosome and proteasome during *Caenorhabditis elegans* aging. For every complex, the abundance variation of each member over the five age groups is reported using the Δ SILAC ratio (difference between the experimental SILAC ratio of a given time point and the SILAC ratio at day 1, left) and the CNV value (right). Each age group has a boxplot (colored as the corresponding time point in the panels A–C of Fig 3) representing the variance between the abundances of the complex members at that given age. Significant changes are marked with a red asterisk (Mann–Whitney test $*P < 0.05$). Related to Fig 3 and Dataset EV6. Boxplots: the horizontal line represents the median of the distribution, the upper and lower limit of the box indicate the first and third quartile, respectively, and whiskers extend 1.5 times the interquartile range from the limits of the box. Values outside this range are indicated as outliers points.

© 2018 The Authors
